# Persistence in a pharmacist-led, same-day PrEP program in Mississippi: a mixed-methods study

**DOI:** 10.1186/s12889-023-16072-1

**Published:** 2023-06-13

**Authors:** Christine M. Khosropour, Taylor Riley, Elise Healy, Kandis V. Backus, Courtney E. Gomillia, Leandro Mena, Khadijra R. Lockwood, Felicia M. Gordon, Arianna R. Means, Lori M. Ward

**Affiliations:** 1 Department of Epidemiology, Hans Rosling Center for Population Health, University of Washington, 3980 15th Ave NE, Seattle, WA 98195 USA; 2 Department of Medicine, Health Sciences Building, University of Washington, 1959 NE Pacific Street, Seattle, WA 98195 USA; 3 Department of Population Health Science, University of Mississippi Medical Center, 2500 N State Street, Jackson, MS39216 USA; 4 Department of Global Health, Hans Rosling Center for Population Health, University of Washington, 3980 15th Ave NE, Seattle, WA 98195 USA

**Keywords:** Pre-exposure prophylaxis, HIV, Retention, Barriers, Mississippi

## Abstract

**Introduction:**

Mississippi has one of the highest rates of HIV in the United States but low PrEP uptake. Understanding patterns of PrEP use can improve PrEP initiation and persistence.

**Methods:**

This is a mixed-method evaluation of a PrEP program in Jackson, Mississippi. Between November 2018-December 2019, clients at high risk for HIV attending a non-clinical testing site were referred to a pharmacist for same-day PrEP initiation. The pharmacist provided a 90-day PrEP prescription and scheduled a follow-up clinical appointment within three months. We linked client records from this visit to electronic health records from the two largest PrEP clinics in Jackson to determine linkage into ongoing clinical care. We identified four distinct PrEP use patterns, which we used for qualitative interview sampling: 1) filled a prescription and linked into care within three months; 2) filled a prescription and linked into care after three months; 3) filled a prescription and never linked into care; and 4) never filled a prescription. In 2021, we purposively sampled patients in these four groups for individual interviews to ascertain barriers and facilitators to PrEP initiation and persistence, using guides informed by the Theory of Planned Behavior.

**Results:**

There were 121 clients evaluated for PrEP; all were given a prescription. One-third were less than 25 years old, 77% were Black, and 59% were cisgender men who have sex with men. One-quarter (26%) never filled their PrEP prescription, 44% picked up the prescription but never linked into clinical care, 12% linked into care at some point after three months (resulting in a gap in PrEP coverage), and 18% linked into care within 3 months. We interviewed 26 of 121 clients. Qualitative data revealed that cost, stigmas related to sexuality and HIV, misinformation about PrEP, and perceived side effects were barriers to uptake and persistence. Individuals’ desire to stay healthy and the support of PrEP clinic staff were facilitators.

**Conclusions:**

The majority of individuals given a same-day PrEP prescription either never started PrEP or stopped PrEP within the first three months. Addressing noted barriers of stigma and misinformation and reducing structural barriers may increase PrEP initiation and persistence.

**Supplementary Information:**

The online version contains supplementary material available at 10.1186/s12889-023-16072-1.

## Introduction

Despite increasing and more widespread availability of oral HIV pre-exposure prophylaxis (PrEP) in the United States (US), PrEP uptake and persistence (i.e., PrEP use over time) have been suboptimal [1, 2]. In 2019, only an estimated 29% of individuals in the US with indications for PrEP were prescribed PrEP, with pronounced disparities by gender, race/ethnicity, and geography [1].

Low PrEP coverage in the US is a result of both low PrEP initiation and poor persistence. Recent meta-analyzed data suggest that about 40% of individuals initiating PrEP in North America will discontinue within six months [2], with real-world clinic-based PrEP programs (i.e., those conducted outside of the context of a research study) observing high drop-off in the first three months after PrEP initiation [3–5]. Barriers to PrEP retention and persistence are multifaceted, including structural, social, and behavioral factors such as an individual’s perception of their ongoing risk of HIV, side effects, provider bias and racism in the healthcare system, and stigma [6–10]. There has been an increasing push to develop strategies to support PrEP persistence and re-initiation [2, 11], but developing these strategies requires an understanding of exactly when individuals disengage from PrEP (e.g., at prescription pick-up, after initiating medication, etc.) and the specific barriers and motivators to initiating and persisting on PrEP at these various steps.

Mississippi has the sixth highest rate of new HIV diagnoses [12] and is one of seven US states identified as a priority focus area for the federal Ending the HIV Epidemic (EHE) initiative [13]. Mississippi’s HIV epidemic is also characterized by profound racial disparities. Nearly three-quarters of new HIV diagnoses are among Black individuals, who represent 39% of the population in Mississippi, and Black individuals have a sixfold higher rate of new diagnosis compared to white individuals [14]. Social determinants of health in Mississippi also have a profound impact on the HIV epidemic. Compared to other US states, Mississippi has the highest poverty rate, the highest percentage of persons experiencing food insecurity, the third highest incarceration rate, the lowest percent of households with broadband internet, and the fifth lowest percent of persons who are insured [15–17].

In 2019, an estimated 21% of individuals in Mississippi with indications for PrEP had been prescribed PrEP [1], which is similar to the national average. However, Mississippi continues to have the lowest “PrEP-to-need ratio” in the US [18, 19], defined as the ratio of the number of PrEP users to the number of people newly diagnosed with HIV in the state. To place this in context, in 2021, Mississippi’s PrEP-to-need ratio indicated that for every 1 person newly diagnosed with HIV, there were 3.56 persons using PrEP. In New York state in 2021, for every 1 person newly diagnosed with HIV, there were 20.28 persons using PrEP.

We have previously described short-term, interim outcomes of a pharmacist-led, same-day PrEP program designed to increase PrEP initiation in Mississippi [20]. Those interim data suggested the potential for low persistence on PrEP, and motivated the present study to continue to follow individuals who initiated PrEP in the program and to better understand reasons for low persistence on PrEP. The goals of the present study were to describe PrEP initiation and persistence of individuals who participated in this program and to examine barriers, motivators, and facilitators to PrEP initiation and persistence.

## Methods

We used an explanatory-sequential (quan->QUAL) mixed-methods study design [21]. We collected and analyzed quantitative and qualitative data in two consecutive phases of the study, wherein the qualitative data were used to explain the quantitative findings. Integration involved connecting the results from the initial quantitative phase to help develop the sampling plan and interview guide for the subsequent qualitative phase. Study procedures and analyses were reviewed and approved by the University of Washington (UW) and University of Mississippi Medical Center (UMMC) Institutional Review Boards (IRB). The UW and UMMC IRBs waived consent for the quantitative analyses of data. For the qualitative analysis of data, verbal consent was obtained from all participants prior to the interview. The UW and UMMC IRBs approved the informed verbal consent procedure.

### Description of PrEP Program

This study utilized data from individuals referred to a same-day PrEP initiation program (“Rapid PrEP”) at Express Personal Health (EPH), a University of Mississippi Medical Center-affiliated, walk-in HIV/STI testing-only clinic in Jackson, Mississippi. Details of the program have been previously described [20]. Briefly, from November 2018 to December 2019 individuals were referred for same-day PrEP initiation if they tested HIV-negative on a rapid HIV test and were cisgender men who have sex with men (MSM), transgender women (TGW), or cisgender women with one of the following: diagnosis with a bacterial STI, contact to a partner with HIV/STI, in an ongoing sexual relationship with an HIV-positive/unknown-status partner, recent injection drug use, or in an ongoing sexual relationship with a MSM. Staff briefly discussed PrEP with eligible clients and referred interested clients to a clinical pharmacist co-located in the same building. The pharmacist (who also served as the PrEP Navigator) provided detailed information about PrEP effectiveness, adherence, and side effects, obtained patients’ medical history, evaluated patients for signs and symptoms of acute HIV, and completed insurance and/or pharmaceutical company medication assistance paperwork. For interested and eligible clients, the pharmacist, operating under a provider protocol [22, 23], sent a 90-day PrEP prescription to clients’ preferred pharmacy for pick-up or shipment from the pharmacy. The pharmacist also scheduled clients’ first clinical PrEP appointment with a PrEP provider within 12 weeks to receive other recommended laboratory tests (i.e., serum creatinine test and hepatitis B screening). The pharmacist provided clients with a phone number which gave clients direct access to the PrEP team by call or text. Clients were encouraged to reach out with non-medically urgent questions, which were responded to during business hours.

### Quantitative methods and analysis

The study population for the quantitative analysis included all individuals referred to the same-day PrEP program between November 2018 and December 2019. All quantitative analyses utilized data collected in the clinic’s electronic PrEP database housed on Research Electronic Data Capture (REDCap) servers [24, 25]. Data were recorded by the program’s pharmacist and included clients’ demographic data, indications for PrEP, insurance and PrEP payment information, and prescription pick-up information. The latter was verified by clinic staff who called pharmacies to confirm the date of prescription pick-up.

We defined four discrete groups that captured the different patterns of PrEP use observed in the quantitative data (Table 1). We defined these groups based on whether or not clients picked up their initial prescription, and if so, whether or not they linked into their first PrEP follow-up visit within 105 days (this time period covered the scheduled clinical appointment within 12 weeks plus a 3-week buffer period in the event that a client re-scheduled their appointment). We focused on the first follow-up visit as a marker for linkage to ongoing PrEP clinical care, because other clinical PrEP programs have noted substantial drop-off within the first three months of PrEP initiation [3–5]. To identify whether individuals subsequently linked into ongoing PrEP clinical care, we used a probabilistic record linkage algorithm [26] to link our program’s REDCap database to electronic databases from the two largest clinical PrEP providers in Mississippi. We followed clients in these clinics’ databases through September 15, 2021 to assess whether or not they discontinued PrEP.

**Table 1:** Groups that capture patterns of PrEP use in the quantitative data

Group	Description
**Group 1**: Linked to ongoing PrEP care	Clients picked up their initial PrEP prescription and linked to a clinical PrEP provider (i.e., attended their first clinical follow-up) within 105 days of prescription pick-up
**Group 2**: Linked to ongoing PrEP care after gap in coverage (started, stopped, and re-started PrEP)	Clients picked up their initial PrEP prescription and linked to a PrEP provider after 105 days from their prescription pick-up, causing a gap in PrEP coverage
**Group 3**: Did not link to ongoing PrEP care (started and stopped PrEP)	Clients picked up their initial PrEP prescription but did not link to a clinical PrEP provider, leading to a stoppage in PrEP
**Group 4**: Never Started PrEP	Clients did not fill their initial PrEP prescription

We compared sociodemographic characteristics, indications for PrEP, insurance status, and payment for PrEP across the groups described in Table 1. Analyses were conducted in R Version 4.0.2 and Stata Version 16.1 (StataCorp LLC, College Station, TX).

### Qualitative methods and analysis

We defined a nested sampling scheme, whereby we categorized clients into one of the four groups described in Table 1. In June–September 2021, we conducted stratified purposive sampling, aiming to interview at least five clients from each group. We attempted to ensure at least two participants in each group were cisgender women and that at least three were Black. This was to ensure that the qualitative study population was reflective of the population receiving same-day PrEP and to ensure adequate representation of cisgender women in the qualitative data. Study staff called potential participants in each PrEP group to describe the study and ask if they were interested in participating in an interview. If someone did not answer or consent, staff reached out to the next potential participant in the respective group. Interviewees underwent informed verbal consent prior to participating in the interview.

Experienced qualitative interviewers conducted one-on-one interviews in-person or via telephone, depending on clients’ preferences. The interviews were guided by a semi-structured interview guide that was informed by both the quantitative data and the Theory of Planned Behavior (TPB) [27, 28]. The TPB posits that the likelihood of an individual engaging in a specific behavior (e.g., PrEP initiation and persistence) is a product of their attitude towards the behavior, the subjective norms around said behavior, and their perceived behavioral control; the TPB has been previously used in the context of PrEP initiation [29, 30]. The guide was adapted for each sampling group (Table 1), resulting in four tailored guides (see Additional file 1 for interview guides). We asked participants about their experiences starting and continuing on PrEP, factors that may have influenced their decision to start and stay on PrEP, and experiences with long-term persistence. Guides were piloted and then adapted based on experiences during piloting. The interviews were digitally recorded and professionally transcribed. The interviewers completed structured debrief notes after each interview. Interviewees were reimbursed $50 for their time and participation in the interview.

To build the codebook for qualitative analyses, we used a combination deductive and inductive approach. An *a priori* list of codes was developed using the TPB. Additional codes were derived from the transcript text (see Additional file 2 for codebook). Transcripts were reviewed for quality and completeness and then uploaded to Dedoose Version 9.0.17 (SocioCultural Research Consultants, LLC, Los Angeles, CA) for qualitative coding. Two primary coders coded all transcripts. Inter-coder reliability meetings were conducted to verify similar code use and modify the codebook as necessary. Coding disagreements were discussed with a third coder serving as tiebreaker. Thematic memos were prepared for each PrEP-use group after coding was complete, organized by TPB domain and non-TPB emerging themes.

## Results

### Quantitative analysis

Between November 2018 and December 2019, there were 121 clients referred to the pharmacist for same-day PrEP; all were ultimately given a PrEP prescription. One-third of clients were less than 25 years old, 77% were Black, 59% were cisgender MSM, and 67% did not have insurance (Table 2). The majority (76%) of planned payment for PrEP was through a pharmaceutical company medication assistance program

**Table 2:** Characteristics of clients presenting for PrEP initiation, overall and by subsequent patterns of PrEP use (*N* = 121)

Characteristic	Total	**Group 1**: Linked to ongoing PrEP care	**Group 2**: Linked to ongoing PrEP care after gap in coverage	**Group 3**: Did not link into ongoing PrEP care	**Group 4**: Never Started PrEP
	**N (Col %)**	**N (Row %)**	**N (Row %)**	**N (Row %)**	**N (Row %)**
**Overall N and Row Percent**	**121 (100)**	**22 (18)**	**15 (12)**	**53 (44)**	**31 (26)**
Age, years					
16–24	40 (33)	11 (28)	6 (15)	17 (43)	6 (15)
25–29	29 (24)	5 (17)	3 (10)	13 (45)	8 (28)
30–35	19 (16)	4 (21)	1 (5)	9 (47)	5 (26)
≥ 35	33 (27)	2 (6)	5 (15)	14 (42)	12 (36)
Gender					
Cisgender men	73 (60)	18 (25)	12 (16)	26 (36)	17 (23)
Cisgender women	46 (38)	4 (9)	2 (4)	26 (57)	14 (30)
Transgender women	2 (2)	(0)	1 (50)	1 (50)	(0)
Race/ethnicity					
Black, non-Hispanic	93 (77)	15 (16)	11 (12)	41 (44)	26 (28)
White, non-Hispanic	23 (19)	4 (17)	4 (17)	10 (43)	5 (22)
Hispanic	4 (3)	3 (75)	(0)	1 (25)	(0)
Other, non-Hispanic	1 (1)	(0)	(0)	1 (100)	(0)
Indication for PrEP^a^					
MSM	71 (59)	18 (25)	12 (17)	25 (35)	16 (23)
Transgender woman	2 (2)	0 (0)	1 (50)	1 (50)	0 (0)
Diagnosed with STI	22 (18)	3 (14)	3 (14)	11 (50)	5 (23)
Contact with partner with HIV/STI	15 (12)	1 (7)	3 (20)	8 (53)	3 (20)
Sexual partner of unknown or serodiscordant HIV status^b^	(0)	(0)	(0)	(0)	(0)
Sexual partner is MSM^b^	(0)	(0)	(0)	(0)	(0)
Insurance status^c^					
Has insurance	40 (33)	12 (30)	6 (15)	16 (40)	6 (15)
Does not have insurance	81 (67)	10 (12)	9 (11)	37 (46)	25 (31)
Payment for PrEP^c^					
Pharmaceutical company patient assistance program	92 (76)	13 (14)	12 (13)	40 (43)	27 (29)
Medicaid	8 (7)	4 (50)	(0)	3 (38)	1 (13)
Private Insurance	20 (17)	5 (25)	2 (10)	10 (50)	3 (15)
Out of pocket	1 (1)	(0)	1 (100)	(0)	(0)

^a^Not mutually exclusive. Data on STI diagnosis, contact to partner with HIV/STI, and information about sexual partners is only available for clients who were referred by health department disease investigation specialists

^b^Indication for cisgender women only

^c^At the time of the initial PrEP initiation visit

Only 18% (*n* = 22) of clients initiated and linked to ongoing PrEP care (Group 1), while 12% (*n* = 15) linked to care after a gap in coverage (Group 2) (Table 2). Around one-quarter (26%; *n* = 31) of clients did not fill their initial PrEP prescription (Group 4) and 44% (*n* = 53) filled their prescription but never attended a subsequent clinical appointment (Group 3). Overall, 87% (*n* = 40/46) of cisgender women either started and stopped PrEP (Group 3) or never started PrEP (Group 4) compared to 59% of cisgender men (*n* = 43/73) (Table 2). A lower proportion of uninsured clients started and stayed on PrEP compared to insured clients (12% vs 30%; Group 1). A higher proportion of those aged 16–24 years started and stayed on PrEP (Group 1) relative to other age groups. We did not note major differences in PrEP use patterns by race/ethnicity.

**Fig. 1:**
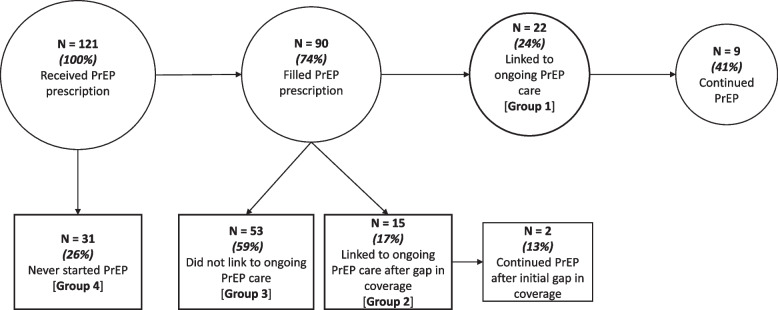
Flow chart of patient outcomes in same-day PrEP program (*N* = 121)

Figure 1 presents a flow chart of patterns of PrEP use; these percentages differ from Table 2 because they are presented as a continuum. Of the 74% of clients who filled their prescription (*n* = 90), 24% (*n* = 22) immediately linked into ongoing PrEP clinical care (Group 1); the median time to linkage to clinical care was 63 days (range: 2–103 days) (data not shown). Only 41% (*n* = 9) of those in Group 1 continued PrEP for another 105 days. Among the 15 individuals who linked into ongoing PrEP care after a gap in coverage (Group 2; 17% of those who filled their prescription), the median time to restarting PrEP was 597 days (range: 145–867 days). Five (16%) of the 31 clients who never filled their PrEP prescription (Group 4) ultimately initiated PrEP later outside of the Rapid PrEP program (data not shown). Their median time from receiving the PrEP prescription from Rapid PrEP to initiating PrEP at another PrEP program was 98 days (range: 4 – 523 days).

#### Qualitative analysis

From June to September 2021 we conducted 26 in-depth interviews with PrEP clients (lasting between 20–60 min each), including six from Group 1, five from Group 2, nine from Group 3 and six from Group 4 (Table 3). Major themes reflect similar and distinctive factors influencing PrEP initiation and persistence across PrEP-use groups, including: (1) individuals’ perceived risk of HIV motivated their initial interest in PrEP; (2) the ease and convenience of the same-day PrEP program enabled participants to access PrEP; (3) perceived and experienced side effects associated with PrEP use were a major barrier for PrEP initiation and persistence; and (4) perceived behavioral control to continue taking PrEP was the primary motivator for persistence and adherence.

**Table 3:** Characteristics of qualitative interview participants, by group (*N* = 26)

**Characteristic**	**Group 1** *N* = 6	**Group 2** *N* = 5	**Group 3** *N* = 9	**Group 4** *N* = 6
	**N (%)**	**N (%)**	**N (%)**	**N (%)**
Age, years				
16–24	1 (17)	2 (40)	4 (44)	0
25–29	1 (17)	1 (20)	1 (11)	0
30–35	2 (33)	0	1 (11)	2 (33)
≥ 35	2 (33)	2 (40)	3 (33)	4 (66)
Gender				
Cisgender men	4 (66)	3 (60)	2 (22)	2 (33)
Cisgender women	1 (17)	2 (40)	7 (77)	4 (66)
Transgender women	1 (17)	0	0	0
Race/ethnicity				
Black, non-Hispanic	4 (66)	4 (80)	7 (77)	5 (83)
White, non-Hispanic	1 (17)	1 (20)	2 (22)	1 (17)
Hispanic	1 (17)	0	0	0
Other, non-Hispanic	0	0	0	0

##### Clients are initially motivated to seek out PrEP based on perceived risk

Across groups, respondents often expressed that their desire to initiate PrEP was due to their perceived risk of acquiring HIV based on their sexual experiences and sexual behavior. For some respondents, this perceived risk was due to their sexual orientation (e.g., being a gay man), while for others the perceived risk was because of their ongoing sexual relationships with partners who were living with HIV.

*“Around the time I was first started PrEP, I was sleeping with a lot of people. [The clinic] was like, it’s best I get on it since I do have a lot of sexual activity, and most of them be new people. So, [the clinic] was like it’s best I try to protect myself*.”

-Black transgender woman, age 28, Group 1

*“I saw many people in the gay bar that we hang out with and we have had sex with, that are positive, or just founding out they're positive. And I would not miss a dose for nothing, because you don’t know who is positive. A lot of people won't tell you they're positive.”*

-White cisgender man, age 58, Group 1

##### Perceived risk (or lack thereof) was also a motivator for PrEP discontinuation

Some individuals in Groups 2 and 3 discontinued PrEP because they felt they no longer needed it. Often, this was because their sexual behaviors and/or relationships had changed, and thus they believed they were no longer at high risk of acquiring HIV.

*“I just didn't really need [PrEP]. Whenever I first started taking it I was really sexually active, and after about that time is when I got into a more serious relationship, and we both got tested and we were fine just to have sex with each other*.” (308)

-Black female, age 28, Group 3

*“I just didn’t really need [PrEP] because I wasn’t sexually active anymore. Because I was taking it as like a precautionary thing and if I wasn’t sexually active I just figured I don’t really need this anymore.”*

-White female, age 22, Group 3

##### Clients who successfully initiated PrEP cited the same-day PrEP program as a primary facilitator

Some respondents in Groups 1, 2, and 3 stated that the PrEP navigator and clinic set-up were positive influential factors in their initiation of PrEP. Specific features discussed included: the pharmacist/navigator’s knowledge and explanation of PrEP, the ability of the pharmacist and staff to make the client feel at ease with initiating PrEP, and the staff’s assistance with the administrative aspects of PrEP initiation (e.g., insurance paperwork, ease in obtaining the prescription).

*“I think that the only reason why I really took the PrEP is because of what [the PrEP navigator] said and how she just was telling me, convincing me how it was and what it was before. I don't think I would have taken it on my own because I think I would just start using the condoms and left it at that.”*

-Black cisgender woman, age 53, Group 2

*“That entire team was very, almost family oriented if you will. Just the whole process of finding out information and seeing my information, filling out the paperwork, everything is set up, the medicine for delivery. That entire team from the receptionist to the last person I saw when doing my paperwork, they were all instrumental in making that process very smooth for me.”*

-Black cisgender man, age 31, Group 1

##### Amongst clients who attempted to initiate PrEP, barriers related to cost and insurance made it challenging to secure medication

Once participants had received their PrEP prescription, some experienced issues at the pharmacy when picking up their prescription, such as being told they needed to pay out-of-pocket because the pharmacy was unable to process their insurance or did not recognize their enrollment in a medication payment assistance program. For respondents in Groups 1–3 (who ultimately succeeded in picking up their prescription), they often reporting reaching out to the PrEP navigator (pharmacist), who helped them access PrEP by working with the pharmacy directly. But for respondents in Group 4, the barriers experienced at the pharmacy largely dissuaded them from starting PrEP.

*“Really, the only problem that I remember having is that at first, they set it up to where something would cover all of the fees, and the pharmacy that I was using…they couldn't get it to where the price was-- I think it ended up being free, but it was a long process. The [PrEP navigator]…had to keep calling up to the pharmacy because they wouldn't fill it, or they would charge me a lot of money. She had given me some kind of insurance that I had to set up. Anyway, she ended up assisting me with it, but it was really hard for the pharmacy to fill it. That was the main problem.”*

-Black cisgender woman, age 28, Group 3

*“The only thing that was challenging, I would have started it but when I remember going to my pharmacy to pick it up and it was too expensive. I just never followed back up. I could have sworn that when I went that it was going to be a little or no payment and then when I got there, it was $300, I was like, "Oh no." [chuckles]”*

-Black cisgender woman, age 45, Group 4

##### Subjective norms around PrEP prevented some clients from initiating or persisting on PrEP

Respondents from all groups reported hearing misinformation about PrEP; a common misconception was that PrEP was for individuals who were living with HIV. Misinformation appeared to dissuade those in Groups 3 and 4 from initiating persisting on PrEP.

*“To be honest, I actually thought it was for people with HIV. Because the way they were saying it on TV, like it helps prevention but when I was reading up on it, it was like it could be treated, too. But it made me skeptical.”*

-Black transgender woman, age 28, Group 1

*“I just took one day because when I had read about it they say it’s like death. Like you could die from taking it.”*

-Black cisgender woman, age 35, Group 3

“*When I left [the PrEP clinic] I went and did my own research, and I didn’t like what I had hear about that PrEP through my research. Then, of course, since then they done had all the errors that went on with it. I was kinda glad I didn’t take it.”*

-Black cisgender man, age 42, Group 4

Respondents also reported hearing stigmatizing assumptions about individuals who use PrEP, including HIV-related stigma. Some respondents who initiated PrEP (Groups 1–3) reported that stigma associated with PrEP led to them keeping their PrEP use private.

*“Well, after learning and being on [PrEP] and I stuck with it because I had the comfort knowing that I was taking an extra safety net to pursue in a safe way the actual sexual life. So, the reason I stayed with it because I got over the stigma of taking a pill to ensure, still today I don’t have HIV. So, that’s a benefit like I don’t have to go down that path of being treated for HIV, having to carry that stigma with the status, which I do understand from the people who are HIV positive point of view. I don’t have that stigma that I am HIV positive. I do understand [inaudible], but I don’t carry that stigma with me personally.”*

-Black cisgender man, age 30, Group 3

##### Perceived and experienced side-effects were a strong motivator for PrEP non-initiation or discontinuation

A main factor that influenced overall PrEP adherence and persistence were perceived or experienced side-effects. While respondents in all groups acknowledged the potential for and, in some cases, experiences of side-effects, the intensity of side-effects and impact on adherence varied. Overall, Group 1 participants seemed less deterred by the potential for, or experience of, PrEP-related side-effects while some respondents in Groups 2 and 3 stopped PrEP primarily because of side-effects.

*“Well, [the PrEP navigator] first let me know that there would be some nausea in the beginning. And I shrugged it off because I’ve seen this. And it just says it’ll make you nauseous, and it never affected me. But this one definitely did. That was the worst nausea I ever experienced in my life just based off of some medicine. But, after that, you know, my body got acclimated”*

-Black cisgender man, age 24, Group 1

*“I really didn't too much like the side effects. That's about the only thing because it wasn't hard taking it once a day. It was just the side effects of my head feeling the way it felt like everything was just spinning all the time. Or me really not having an appetite to eat or me feeling constant nausea.”*

-Black cisgender woman, age 22, Group 3

*“The after effect is not worth it…I was gonna take it but after I read about it and it was on the TV about it, I just say no. I’m not trying to get myself messed up off some pills.”*

-Black cisgender woman, age 35, Group 4

##### Persisting on PrEP was influenced by perceived behavioral control

Respondents from Groups 1–3 all expressed that the motivation to stay on PrEP was tied to perceived behavioral control (i.e., their ability to remember and take the medication as prescribed) rather than external factors.

*“One person can only do so much but it’s – someone has to take the responsibility ultimately of their own actions. I mean, you could lead anyone to water but you can't make them drink the water. You could want and have for anyone but if they don't wanna go out and do it and they want it and achieve it for themselves, they will never understand that the value behind that.”*

-Black male, age 31, Group 1

*“…it wasn't too hard to just start the medicine. Like I said, you have to have an open mind and you have to know it's a direct thing with taking your medicine. You have to make sure you're reminding yourself to take it. You have to take the responsibility to set reminders and things like it. It's really up to the person. It was easy for me being that I was already organized and I knew I had to get to that for myself.*

-Black male, age 21, Group 2

## Discussion

In this mixed-methods evaluation of a same-day, pharmacist-led PrEP program in Mississippi, we observed high uptake of an initial PrEP prescription but relatively low clinical follow-up and persistence on PrEP. Clients who did not persist on PrEP or never filled a PrEP prescription often cited structural barriers (e.g., cost), side effects, and changes in HIV risk as barriers to staying on PrEP while perceived behavioral control (i.e. confidence in ability to persist on PrEP) was a main factor influencing persistence for those who continued on PrEP. Stigma and misinformation were repeatedly cited as challenges to initiating or persisting on PrEP. Our findings demonstrate how same-day PrEP programs can support PrEP initiation, but also reveal the challenges in promoting PrEP persistence.

Our quantitative findings underscore the critical need to improve retention and persistence on PrEP. In this 13-month period, we successfully provided a same-day PrEP prescription to 121 individuals at high risk of HIV in a high HIV morbidity jurisdiction. Yet those positive findings are overshadowed by the fact that 70% of individuals who received a PrEP prescription either never filled their prescription (26%; Group 4) or never returned to the clinic after filling the initial prescription (44%; Group 3). Although several other studies in the Southern US have noted suboptimal rates of PrEP persistence, the level of discontinuation in our study population is high relative to what has been previously observed. In a PrEP demonstration project in three US cities, only 11% of patients who received a PrEP prescription never returned to the clinic (analogous to Group 3) [31]. Among a cohort of young Black MSM in Atlanta, Serota and colleagues [32] noted that 69% of PrEP initiators ever stopped PrEP, but the median time to first discontinuation was 159 days, whereas, in our study population, 44% of individuals (Group 3) discontinued PrEP after their initial 90-day PrEP prescription. In clinical settings in Atlanta [3], Mississippi, Missouri and Rhode Island [4], approximately 50%-73% of patients returned for their 3-month visit (the analogous percentage in our study was 18% [Group 1]). It is also notable that 26% of individuals in our study who left the clinic with a PrEP prescription in-hand never filled their prescription. Other clinic-based studies of PrEP provision have found that between 12 to 19% of individuals never start PrEP after receiving their prescription [3, 4, 33].

Given the high rates of discontinuation observed in this PrEP program, the qualitative findings provide particularly invaluable insights. We observed four key themes from the interviews that can be used to improve this and other same-day PrEP programs. First, clients’ initial interest in starting PrEP seemed to be driven largely by their perceived risk of acquiring HIV, but many clients noted that the same-day PrEP program itself was a motivator for initiation. This highlights the importance of programs to provide adequate and appropriate education so that individuals are aware of HIV risk, but doing so in a comfortable and non-threatening environment.

Second, many clients remarked on challenges they had securing medication at the pharmacy and/or the perceived cost of the medication. Although in our program the pharmacist provided the prescription, the client still had to go to a pharmacy to pick up the medication and sometimes encountered barriers when they arrived at the pharmacy. In some cases, this prohibited individuals from starting PrEP. We postulated that barriers encountered at the pharmacy may have been due to the fact that PrEP use was not widespread in Mississippi at this time, but pharmacy challenges have also been noted as barriers to PrEP use in New York [34]. PrEP-related training specifically for pharmacists may reduce some of these barriers. Further, other qualitative studies in Mississippi and across the US have emphasized the need to reduce the number of steps in securing medication [10, 35, 36], and our findings align with those studies. Importantly, our program did attempt to reduce structural barriers (e.g., assist with required medication assistance paperwork) to PrEP initiation – a key step to promoting PrEP uptake [6] – but the medication pick-up still posed a barrier. To eliminate this barrier, some clinics may be willing and able to keep generic PrEP medication on-site to eliminate an additional stop at a pharmacy for the initial pick-up.

Third, qualitative interviews revealed that stigma and misinformation were pervasive. Stigma associated with HIV, PrEP use, and being gay were repeatedly cited when discussing PrEP use. Although this may have resulted in individuals being less likely to persist on PrEP, these stigmas also often led to individuals not wishing to disclose their PrEP use to others. Misinformation about PrEP led some individuals to never initiate PrEP or stop taking it if they believed that the medication was not appropriate for them. These barriers, driven by perceived subjective norms, have been noted in other studies [6, 7, 10, 34, 35, 37, 38], and have been linked to a willingness (or lack thereof) to use PrEP. Here we found that misinformation about PrEP directly resulted in individuals not wishing to start PrEP or stay on PrEP. These finding underscores a critical need to develop messaging that directly combats stigma PrEP misinformation. There is widespread HIV stigma and homophobia in Mississippi, [39] and understanding the misinformation that is predominate in the community is key for PrEP clinic staff to be able to provide factual counter-messages to the misinformation (e.g., provide clarifying messages about statements that are misinterpreted from TV advertisements), and for clinics to be able to advertise PrEP services in a way that directly combats that misinformation.

Fourth, we found that many clients stopped PrEP because of perceived or experienced side effects or when they believed they were at low risk of HIV. However, recent longitudinal studies of individuals on PrEP have observed relatively high rates of HIV among former PrEP users who stopped PrEP [40]. Our program and others can improve messaging about side effects and/or encourage clients to reach out to the clinic if they experience side effects to discuss the decision to stop medication before actually doing so. We also believe that programs should continue to educate clients about potential HIV risk and encourage clients to re-evaluate their HIV risk with a PrEP provider or PrEP clinic staff prior to stopping PrEP, in order to make an informed decision prior to stopping PrEP.

Together, the quantitative and qualitative findings highlight the importance of, and need for, interventions delivered at the time of PrEP initiation and beyond that can decrease structural barriers and promote persistence in PrEP care. In addition to the “lessons learned” described above, interventions such as mHealth and texting interventions, contingency management interventions, and patient navigation interventions may improve PrEP prescription pick-up and/or persistence on PrEP [41–44]. Further, we believe that building a PrEP program that is adaptable and can meet the needs of different clients is paramount. We originally sought to target our same-day PrEP program to MSM, but later expanded to encourage cisgender women. We also continued to communicate with clients who had linked into clinical care, as some wanted to continue to communicate and/or have visits with the pharmacist in an ongoing capacity. Implementing these interventions and adaptable programs as routine practice in clinical PrEP settings is a necessary next-step to improving PrEP persistence. However, our findings also suggest that ongoing community education and outreach to combat stigma and misinformation and to normalize PrEP use are vital to improve PrEP initiation and persistence, particularly in the Deep South.

Our findings are also informative in their revelation of the critical limitations of quantitative data to describe patterns of PrEP use. For example, individual interviews indicated that some individuals categorized in Group 3 never actually took any PrEP despite picking up their prescription, while others did link into ongoing clinical care but not in the two clinical settings for which data were available. Additionally, our differentiation of Groups 2 and 3 was based off of our administrative closure of the quantitative dataset, and individuals in Group 3 could have linked into clinical care after we closed the dataset. Indeed, qualitative analyses revealed that these two groups were thematically quite similar. Further, although our quantitative data examined persistence and retention based on whether or not people returned to the clinic for their first and subsequent appointments, many interviewees focused more on adherence to medication (i.e. taking medication) instead of returning to the clinic for visits, and the first clinical follow-up appointment after a PrEP prescription was not a particularly notable event for many clients. These important distinctions between quantitative and qualitative data are a reminder that quantitative data are not able to fully capture the dynamics of PrEP use nor the reasons behind those dynamics. Studies relying solely on medical records to quantify PrEP persistence should be aware of these critical limitations.

This study has several strengths, including the explanatory-sequential mixed methods design, systematic data collection with documented PrEP prescription pick-up, and our linkage of programmatic data with two of the largest clinical PrEP providers to track clients’ patterns of clinic engagement over time. However, there are also several limitations. First, as noted above, our categorization of clients into PrEP-use groups, which was based on the quantitative data, was imperfect and may have miscategorized clients resulting in incorrect estimates of persistence. Second, although we had access to the clinic records of two large PrEP programs, we did not have access to all PrEP providers in the area and it is possible that some individuals in our program were indeed in clinical care elsewhere but we were unable to capture this. Third, the quantitative data captures only clinic visits to renew prescriptions and does not capture actual adherence to PrEP. It is possible that some individuals used PrEP intermittently, on a dosing schedule that may still be efficacious for HIV prevention [45] and allow them to “extend” their prescription past a 90-day period. Fourth, we did not specifically ask study participants about whether distance to ongoing PrEP clinical care was a barrier. Of note, we did probe about transportation and did not find that to be a commonly-noted barrier, but we did not explicitly ask about distance. Fifth, the TPB does not specifically address structural factors; however, we used an inductive (driven by data) approach to coding in addition to a deductive approach (driven by TPB) to account for this. Finally, these results are from a single setting in Jackson, Mississippi and may not be generalizable, though both our quantitative and qualitative findings generally align with what has been observed in other studies.

## Conclusions

In conclusion, we observed high rates of PrEP discontinuation in this same-day PrEP program, with noted barriers of securing medication, change in perceived HIV risk, and stigma and misinformation influencing PrEP initiation and persistence. However, the program appeared to facilitate rendering of the initial prescription and was able to mitigate some barriers to starting PrEP. Interventions focused on enhancing persistence on PrEP, helping clients understand their own HIV risk, supporting re-initiation of PrEP for those who have stopped, building confidence in clients’ PrEP usage, and facilitating PrEP re-starts should be prioritized as we enter the next phase of the EHE initiative.

## Supplementary Information

**Additional file 1.** 

**Additional file 2.** 

## Data Availability

Quantitative and qualitative data are not publicly archived but de-identified data can be made available to interested parties upon request by contacting Christine Khosropour at ckhosro@uw.edu.

## Abbreviations

PrEP: Pre-exposure prophylaxis
US: United States
EHE: Ending the HIV Epidemic
EPH: Express Personal Health
MSM: Men who have sex with men
TGW: Transgender women
