# Supplementary material for: Persistence in a pharmacist-led, same-day PrEP program in Mississippi: a mixed-methods study
Source: BMC Public Health. 2023 Jun 13;23:1130. doi: 10.1186/s12889-023-16072-1 (PMC10262591; doi:10.1186/s12889-023-16072-1)
Supplement: Supplementary file 1 — Additional file 1. [file 12889_2023_16072_MOESM1_ESM.docx]

**Supplementary File 1: Qualitative Interview Guide for RapidPrEP Evaluation**

**INTRODUCTORY SCRIPT**

**For phone interviews (if calling the client), begin with:** Hello, this is [your name] calling from the University of Mississippi Medical Center. I am calling about the PrEP navigation services research study that you agreed to participate in. We had set up this time to conduct the interview over the phone. Is this still a good time for you?

Thank you for taking the time to speak with us today. This interview should take about an hour [30 minutes (*for non-PrEP users*)] of your time. We’d like to take a minute to introduce ourselves, and the project.

**For Zoom interviews, begin with:** Thank you for taking the time to speak with us today for this research study. This interview should take about an hour [30 minutes (*for non-PrEP users*)] of your time. We’d like to take a minute to introduce ourselves, and the project.

*[Interviewer introduce, note taker introduce]*

We are working on a project to understand opportunities to make it easier for people who are interested in PrEP to access it. Specifically, we will be asking about PrEP navigation programs that help people start PrEP quickly. When we refer to a “PrEP Navigator”, that may be someone you worked with at the medical mall who discussed PrEP with you and helped you get started with it. We’d like to learn about your experiences, and what made it easier or harder to access these PrEP services. We will be using these findings to make recommendations for improving PrEP navigation services in Mississippi .

Before I continue, I’d like to pause first to see if you have any questions about the overall purpose of this project?

Before we begin, I want to make sure it’s clear that this conversation is completely confidential, and your name as well as any personal information that you provide will not be used or shared in any way. Any quotes or information learned during this interview will be de-identified, meaning we will not mention you or specific organizations that you have received services from anywhere in our findings. With your permission, we would like to record this conversation, which will produce a transcription of this interview. We will delete the recording after checking the transcription and the transcription data will be stored separately from your name and contact information. Only team members will have access to the recording and transcription, and we will securely store them. Participating in this interview is completely voluntary, and you can end the interview at any time. Refusal to participate will not have any negative impact on any benefits or services you might be receiving from Mississippi Medical Center.

As a reminder, you will receive a $50 Wal-Mart gift card by email for participating in this interview.

Do you have any questions about participating in this interview?

Yes (provide answers) No

I just want to remind you that participating in this interview is completely optional and you may choose to not respond to a question or stop the interview at any time. Do you agree to participate in this interview?

Yes No

Do we have your permission to record this interview?

Yes (hit record) No

Thank you, you will see a notification from Zoom pop-up now, please accept that this Zoom conversation will be recorded.

**
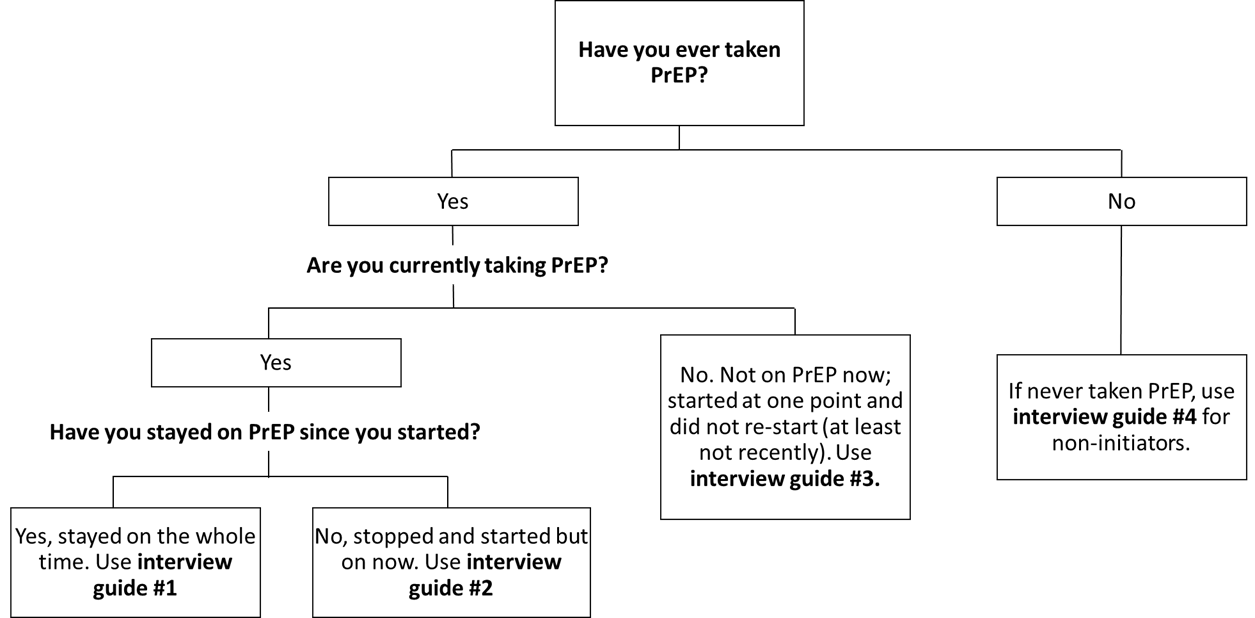
*Once the interview begins, start by using the question flow chart below to determine which group the client falls into.***

Please ask the questions below, relevant to the client’s group. Afterwards, please go to the script relevant to each client’s group.

| **#** | **Groups** | **Questions** |
| --- | --- | --- |
| **1** | Groups 1, 2, and 3 | When did you first start taking PrEP? |
| **2** | Group 2 (on PrEP now, but has started and stopped) | How many times have you stopped taking PrEP? |
|  |  | Last time you **stopped** taking PrEP, for about how long did you **stop** taking it? |
|  |  | What was the reason for stopping, last time you stopped? |
| **3** | Group 3 (stopped PrEP and never restarted) | How long did you take PrEP before you stopped? |
|  |  | When did you stop taking PrEP |
|  |  | Why did you stop taking PrEP? |

**GROUP 1 INTERVIEW GUIDE: Started on PrEP and stayed on the whole time**

***I’d like to begin by learning about your experiences being offered PrEP. The first few questions relate to when you were offered PrEP from Kandis, the pharmacist who was working as a PrEP Navigator, a couple of years ago.***

1. What had you heard about PrEP before speaking with Kandis? (*TPB construct: Attitude)*
2. At the time, why were you interested in starting PrEP? (*TPB construct: Behavior intention*)
3. Did you know anyone who was taking PrEP at that time? (*TPB construct: Subjective norms*)
   1. If yes: What is your relationship to this person?
   2. If yes: Did this person influence your decision to start PrEP in any way?
   3. If no: Did that influence your comfort in starting PrEP, in any way?

***I’d like to learn more about your experiences when you and Kandis discussed whether you would start PrEP.***

1. Can you briefly describe what it was like to start working with the rapid PrEP navigator, Kandis?
   1. How did Kandis first make contact with you?
   2. Was it clear what services Kandis would be providing you? What were they?
2. Can you tell me about your experiences starting PrEP? What was easy about the experience?
   1. (**Example that can be provided only if interviewee does not understand the question:** navigator doing paperwork, navigator checking in, getting Rx the same day as the HIV test) (*TPB construct: Perceived behavioral control)*
   2. Please tell me more. Why do you think this step/process was easy for you?
3. What was challenging about the experience of starting PrEP? (*TPB construct: Perceived behavioral control)*
   1. If yes: Thanks, please tell me more about this. Why do you think this was so challenging?
   2. If nothing was a challenge: What do you think might be challenging about this process for others?
   3. Did you experience any difficulties accessing the pharmacy or picking up your prescription?
4. What would have made it easier for you to start PrEP? (*TPB construct: Perceived behavioral control)*
   1. Was there anything that that the clinic could have done to make it easier for you to start PrEP? (**Example that can be provided only if interviewee does not understand the question**: more check-ins, mailing prescription, etc)
   2. Is there anything the PrEP navigator, Kandis, could have done to make it easier for you to start PrEP?

***Now, I’d like to ask some questions about your experiences continuing to take PrEP over time***

1. What helped you stay on PrEP? (*TPB construct: Perceived behavioral control)*
   1. Did the clinic or program do anything to help you stay on PrEP? (If yes, please explain)
   2. Is there anything the PrEP navigator, Kandis, or other people who worked for the clinic, could have done to make it easier for you to stay on PrEP?
2. What has made it difficult to stay on PrEP? (*TPB construct: Perceived behavioral control)*
   1. Were there any factors related to the clinic that made it difficult to stay on PrEP? (**Example that can be provided only if interviewee does not understand the question:** For example, was it hard to get an appointment or to come in every three months?)
   2. Thanks, can you tell me more about this?
   3. Has access to transportation influenced your PrEP use?
      1. If no, why hasn’t this been an issue for you? What transportation do you use?
      2. If yes, how so?
   4. Has cost or insurance influenced your PrEP use?
      1. If not, why hasn’t this been an issue for you?
      2. If yes, how so?
3. How did the opinions of your family and friends influence your PrEP use?
   1. Tell me more: did you feel supported by your friends and family in your PrEP use? Why or why not?
4. What do you think would make it easier for you to stay on PrEP in the future? (*TPB construct: Intention)*
   1. Is there anything the clinic could do to make it easier for you to stay on PrEP? (**Example that can be provided only if interviewee does not understand the question**: For example, appointment frequency could be decreased, changes can be made so you don’t have to come in person, etc.).
   2. *Follow-up if they don’t intend to stay on PrEP*: Can you tell me more about why you aren’t planning to stay on PrEP?
5. Have you had any PrEP follow-up visits by phone or video?
   1. If yes, how have phone/video visits affected your PrEP experience?
      1. Probe: Has it made it easier to attend your PrEP related visits?
   2. If no, do you think that doing visits over the phone/video would help you stay on PrEP? Please explain.
      1. Probe: How would not having to travel to the clinic impact your ability to stay on PrEP?

***Thank you so much for your time today. I just have two more questions as we wrap-up, to see if you have any further feedback for us.***

1. If a friend was interested in PrEP, what would your advice be for them?
2. What is your advice to the clinic about how to improve PrEP services in the future?
   1. Probe: Can you elaborate?

**GROUP 2 INTERVIEW GUIDE: Started and stopped PrEP, but on now**

***I’d like to begin by learning about your experiences being offered PrEP. The first few questions relate to when you were offered PrEP from Kandis, the pharmacist who was working as a PrEP Navigator, a couple of years ago.***

1. What had you heard about PrEP before speaking with Kandis? (*TPB construct: Attitude)*
2. At the time, why were you interested in starting PrEP? (*TPB construct: Behavior intention*)
3. Did you know anyone who was taking PrEP at that time? (*TPB construct: Subjective norms*)
   1. If yes: What is your relationship to this person?
   2. If yes: Did this person influence your decision to start PrEP in any way?
   3. If no: Did that influence your comfort in starting PrEP, in any way?

***I’d like to learn more about your experiences when you and Kandis discussed whether you would start PrEP.***

1. Can you briefly describe what it was like to start working with the rapid PrEP navigator, Kandis?
   1. How did Kandis first make contact with you?
   2. Was it clear what services Kandis would be providing you? What were they?
2. Can you tell me about your experiences starting PrEP? What was easy about the experience?
   1. (**Example that can be provided only if interviewee does not understand the question:** navigator doing paperwork, navigator checking in, getting Rx the same day as the HIV test) (*TPB construct: Perceived behavioral control)*
   2. Please tell me more. Why do you think this step/process was easy for you?
3. What was challenging about the experience of starting PrEP? (*TPB construct: Perceived behavioral control)*
   1. If yes: Thanks, please tell me more about this. Why do you think this was so challenging?
   2. If nothing was a challenge: What do you think might be challenging about this process for others?
   3. Did you experience any difficulties accessing the pharmacy or picking up your prescription?
4. What would have made it easier for you to start PrEP? (*TPB construct: Perceived behavioral control)*
   1. Was there anything that that the clinic could have done to make it easier for you to start PrEP? (**Example that can be provided only if interviewee does not understand the question**: more check-ins, mailing prescription, etc)
   2. Is there anything the PrEP navigator, Kandis, could have done to make it easier for you to start PrEP?

***Now, I’d like to ask some questions about your experiences continuing to take PrEP over time***

1. What helped you stay on PrEP? (*TPB construct: Perceived behavioral control)*
   1. Did the clinic or program do anything to help you stay on PrEP? (If yes, please explain)
   2. Is there anything the PrEP navigator, Kandis, or other people who worked for the clinic, could have done to make it easier for you to stay on PrEP?
2. What has made it difficult to stay on PrEP? (*TPB construct: Perceived behavioral control)*
   1. Were there any factors related to the clinic that made it difficult to stay on PrEP? (**Example that can be provided only if interviewee does not understand the question:** For example, was it hard to get an appointment or to come in every three months?)
   2. Thanks, can you tell me more about this?
   3. Has access to transportation influenced your PrEP use?
      1. If no, why hasn’t this been an issue for you? What transportation do you use?
      2. If yes, how so?
   4. Has cost or insurance influenced your PrEP use?
      1. If not, why hasn’t this been an issue for you?
      2. If yes, how so?
3. How did the opinions of your family and friends influence your PrEP use?
   1. Tell me more: did you feel supported by your friends and family in your PrEP use? Why or why not?
4. What do you think would make it easier for you to stay on PrEP in the future? (*TPB construct: Intention)*
   1. Is there anything the clinic could do to make it easier for you to stay on PrEP? (**Example that can be provided only if interviewee does not understand the question**: For example, appointment frequency could be decreased, changes can be made so you don’t have to come in person, etc.).
   2. Follow-up if they don’t intend to stay on PrEP: Can you tell me more about why you aren’t planning to stay on PrEP?
5. Have you had any PrEP follow-up visits by phone or video?
   1. If yes, how have phone/video visits affected your PrEP experience?
      1. Probe: Has it made it easier to attend your PrEP related visits?
   2. If no, do you think that doing visits over the phone/video would help you stay on PrEP? Please explain.
      1. Probe: How would not having to travel to the clinic impact your ability to stay on PrEP?

***Thank you so much for your time today. I just have two more questions as we wrap-up, to see if you have any further feedback for us.***

1. If a friend was interested in PrEP, what would your advice be for them?
2. What is your advice to the clinic about how to improve PrEP services in the future?
   1. Probe: Can you elaborate?

**GROUP 3 INTERVIEW GUIDE: Started PrEP but no longer on**

***I’d like to begin by learning about your experiences being offered PrEP. The first few questions relate to when you were offered PrEP from Kandis, the pharmacist who was working as a PrEP Navigator, a couple of years ago.***

1. What had you heard about PrEP before speaking with the navigator? (*TPB construct: Attitude)*
2. At the time, why were you interested in starting PrEP? (*TPB construct: Behavior intention*)
3. Did you know anyone who was taking PrEP at that time? (*TPB construct: Subjective norms*)
   1. If yes: What is your relationship to this person?
   2. If yes: Did this person influence your decision to start PrEP in any way?
   3. If no: Did that influence your comfort in starting PrEP, in any way?

***I’d like to learn more about your experiences when you and Kandis discussed whether you would start PrEP.***

1. Can you briefly describe what it was like to start working with the rapid PrEP navigator, Kandis?
   1. How did the navigator first make contact with you?
   2. Was it clear what services the navigator would be providing you? What were they?
2. Can you tell me about your experiences starting PrEP? What was easy about the experience?
   1. (**Example that can be provided only if interviewee does not understand the question:** navigator doing paperwork, navigator checking in, getting Rx the same day as the HIV test) (*TPB construct: Perceived behavioral control)*
   2. Please tell me more. Why do you think this step/process was easy for you?
3. What was challenging about the experience of starting PrEP? (*TPB construct: Perceived behavioral control)*
   1. If yes: Thanks, please tell me more about this. Why do you think this was so challenging?
   2. If nothing was a challenge: What do you think might be challenging about this process for others?
   3. Did you experience any difficulties accessing the pharmacy or picking up your prescription?
4. What would have made it easier for you to start PrEP? (*TPB construct: Perceived behavioral control)*
   1. Was there anything that that the clinic could have done to make it easier for you to start PrEP? (**Example that can be provided only if interviewee does not understand the question**: more check-ins, mailing prescription, etc)
   2. Is there anything the PrEP navigator, Kandis, could have done to make it easier for you to start PrEP?

***Now, I’d like to ask some questions about your experiences continuing to take PrEP over time***

1. What helped you stay on PrEP? (*TPB construct: Perceived behavioral control)*
   1. Did the clinic or program do anything to help you stay on PrEP? (If yes, please explain)
   2. Is there anything the PrEP navigator, Kandis, or other people who worked for the clinic, could have done to make it easier for you to stay on PrEP?
2. What has made it difficult to stay on PrEP? (*TPB construct: Perceived behavioral control)*
   1. Were there any factors related to the clinic that made it difficult to stay on PrEP? (**Example that can be provided only if interviewee does not understand the question:** For example, was it hard to get an appointment or to come in every three months?)
   2. Thanks, can you tell me more about this?
   3. Has access to transportation influenced your PrEP use?
      1. If no, why hasn’t this been an issue for you? What transportation do you use?
      2. If yes, how so?
   4. Has cost or insurance influenced your PrEP use?
      1. If not, why hasn’t this been an issue for you?
      2. If yes, how so?
   5. Were there any issues when the navigator left, or with switching your care between different providers?
3. How did the opinions of your family and friends influence your PrEP use?
   1. Tell me more: did you feel supported by your friends and family in your PrEP use? Why or why not?
4. What do you think would make it easier for you to stay on PrEP in the future? (*TPB construct: Intention)*
   1. Is there anything the clinic could do to make it easier for you to stay on PrEP? (**Example that can be provided only if interviewee does not understand the question**: For example, appointment frequency could be decreased, changes can be made so you don’t have to come in person, etc.).
   2. Follow-up if they don’t intend to stay on PrEP: Can you tell me more about why you aren’t planning to stay on PrEP?
5. Have you had any PrEP follow-up visits by phone or video?
   1. If yes, how have phone/video visits affected your PrEP experience?
      1. Probe: Has it made it easier to attend your PrEP related visits?
   2. If no, do you think that doing visits over the phone/video would help you stay on PrEP? Please explain.
      1. Probe: How would not having to travel to the clinic impact your ability to stay on PrEP?

***Thank you so much for your time today. I just have two more questions as we wrap-up, to see if you have any further feedback for us.***

1. If a friend was interested in PrEP, what would your advice be for them?
2. What is your advice to the clinic about how to improve PrEP services in the future?
   1. Probe: Can you elaborate?

**GROUP 4 INTERVIEW GUIDE: Never started PrEP**

***I’d like to begin by learning about your experiences being offered PrEP. The first few questions relate to when you were offered PrEP from Kandis, the pharmacist who was working as a PrEP Navigator, a couple of years ago.***

1. What had you heard about PrEP before speaking with the navigator? (*TPB construct: Attitude)*
2. At the time, why were you interested in starting PrEP? (*TPB construct: Behavior intention*)
3. Did you know anyone who was taking PrEP at that time? (*TPB construct: Subjective norms*)
   1. If yes: What is your relationship to this person?
   2. If yes: Did this person influence your decision to start PrEP in any way?
   3. If no: Did that influence your comfort in starting PrEP, in any way?

***I’d like to learn more about your experiences when you and Kandis discussed whether you would start PrEP.***

1. Can you briefly describe what it was like to start working with the rapid PrEP navigator, Kandis?
   1. How did the navigator first make contact with you?
   2. Was it clear what services the navigator would be providing you? What were they?
2. What was challenging about the experience of starting PrEP? (*TPB construct: Perceived behavioral control)*
   1. Did you experience any difficulties accessing the pharmacy or picking up your prescription?
      1. Probe: How do you think this could be improved?
   2. Did you experience any difficulties with the navigator, or other providers themselves?
      1. Prove: How do you think this could be improved?
   3. Did you have any unanswered questions, that made you uncomfortable starting PrEP?
3. What would have made it easier for you to start PrEP? (*TPB construct: Perceived behavioral control)*
   1. Probe: Was there anything that that the clinic could have done to make it easier for you to start PrEP? (**Example that can be provided only if interviewee does not understand the question**: more check-ins, mailing prescription, etc)
   2. Is there anything the PrEP navigator, Kandis, could have done to make it easier for you to start PrEP?
4. Were you worried at all about what your friends or family might think if you started PrEP?
   1. *If yes:* Please explain, whose opinions mattered to you? What were your concerns?
   2. *If no:* That is great to hear. This is an issue for many people. Why do you think this was not a major challenge for you?

***Thank you so much for your time today. I just have two more questions as we wrap-up, to see if you have any further feedback for us.***

1. If a friend was interested in PrEP, what would your advice be for them?
2. What is your advice to the clinic about how to improve PrEP services in the future?
   1. Probe: Can you elaborate?
