# Supplementary material for: Persistence in a pharmacist-led, same-day PrEP program in Mississippi: a mixed-methods study
Source: BMC Public Health. 2023 Jun 13;23:1130. doi: 10.1186/s12889-023-16072-1 (PMC10262591; doi:10.1186/s12889-023-16072-1)
Supplement: Supplementary file 2 — Additional file 2. [file 12889_2023_16072_MOESM2_ESM.docx]

**Supplementary File 2**. Codebook for RapidPrEP qualitative analysis

| CODE | TPB CONSTRUCT | DESCRIPTION | INCLUSION & EXCLUSION CRITERIA |
| --- | --- | --- | --- |
| PrEP Initiation | Behavioral Intention | Individual’s perception of how difficult or easy it was or would be for them to start PrEP and their willingness to start PrEP |  |
| PrEP Adherence | Behavioral Intention | Individual’s perception of how difficult or easy it was or would be for them to adhere to PrEP |  |
|  |  |  |  |
| PrEP belief | Attitude | Individual’s perspective on using PrEP and if they are favorable or unfavorable towards PrEP. |  |
| PrEP motivation | Attitude | Individual’s statements on factors that influence their motivation to initiate or persist on PrEP |  |
| Misinformation | Attitude | Statements about rumors or beliefs about PrEP that are not scientifically correct, and how they affect PrEP initiation or persistence. |  |
| Side-effects | Attitude | Statements about perceived negative side effects, or the absence of side effects, that interviewees heard of and/or experienced related to PrEP use |  |
|  |  |  |  |
| Social pressure | Subjective Norms | Individual’s perceptions about the normative PrEP behaviors within a community (beliefs about whether most people use/don’t use PrEP) or perception about whether most people approve or disprove of PrEP use and how this impacts their decision to use PrEP. |  |
| PrEP stigma | Subjective Norms | Individual’s perceptions ofPrEP stigma (negative assumptions and beliefs about people who use PrEP) or the absence of stigma influence PrEP initiation or retention |  |
| HIV stigma | Subjective Norms | Individual’s perceptions of how stigma (negative assumptions and beliefs about people who are living with HIV) or the absence of stigma influence PrEP initiation or retention | (In some instances, may be difficult to differentiate from PrEP stigma. Double code when necessary) |
| Sexuality stigma | Subjective Norms | Individual’s perception of how positive or negative assumptions about people who are LGBTQAI+ affects PrEP initiation and/or adherence |  |
|  |  |  |  |
| PrEP Self-efficacy | Behavioral Control | Perceived personal capacity to start and/or adhere and take PrEP as directed. |  |
| Perceived Tolerance | Behavioral Control | Perceived physical capacity to tolerate PrEP and its impact on starting and/or adhering and taking PrEP as directed. |  |
| Structural Racism | Behavioral Control | Racism embedded in society and especially the healthcare system that affects an individuals’ access to or interest in PrEP |  |
|  |  |  |  |
| Gender | Cross-cutting | Aspects of navigating healthcare or accessing PrEP that differ depending on the interviewee’s gender |  |
| Sexuality | Cross-cutting | Aspects of navigating healthcare or accessing PrEP that differ depending on the interviewee’s sexuality |  |
| Transportation | Cross-cutting | Transportation as a facilitator or barrier for initiating and/or adhering to PrEP |  |
| Pharmacy | Cross-cutting | Aspects of the pharmacy as a facilitator or barrier for initiating and/or adhering to PrEP |  |
| Clinic | Cross-cutting | Aspects of the clinic as a facilitator or barrier for initiation and/or adhering to PrEP |  |
| Good quote | Cross-cutting | Any quote that is particularly well stated or note-worthy |  |

TPB, Theory of Planned Behavior
